# The Impact of *Drosophila Awd/NME1/2* Levels on Notch and Wg Signaling Pathways

**DOI:** 10.3390/ijms21197257

**Published:** 2020-10-01

**Authors:** Giulia Serafini, Giorgia Giordani, Luca Grillini, Davide Andrenacci, Giuseppe Gargiulo, Valeria Cavaliere

**Affiliations:** 1 Dipartimento di Farmacia e Biotecnologie, Alma Mater Studiorum Università di Bologna, 40126 Bologna, Italy; serafini@mpi-cbg.de (G.S.); giorgia.giordani3@unibo.it (G.G.); luca.grillini2@studio.unibo.it (L.G.); 2 Max Planck Institute of Molecular Cell Biology and Genetics, 01307 Dresden, Germany; 3 Unit of Bologna, CNR Institute of Molecular Genetics “Luigi-Luca Cavalli-Sforza”, 40136 Bologna, Italy; dandrena@area.bo.cnr.it; 4 IRCCS Istituto Ortopedico Rizzoli, 40136 Bologna, Italy

**Keywords:** *Awd/NME*, Wg signaling, Notch signaling, RNAi, hypomorph, chromosomal instability

## Abstract

Awd, the *Drosophila* homologue of *NME1/2* metastasis suppressors, plays key roles in many signaling pathways. Mosaic analysis of the null *awd^J2A4^* allele showed that loss of *awd* gene function blocks Notch signaling and the expression of its target genes including the Wingless (Wg/Wnt1) morphogen. We also showed that RNA interference (RNAi)-mediated *awd* silencing (*awdi*) in larval wing disc leads to chromosomal instability (CIN) and to Jun amino-terminal kinases (JNK)-mediated cell death. Here we show that this cell death is independent of p53 activity. Based on our previous finding showing that forced survival of *awdi*-CIN cells leads to aneuploidy without the hyperproliferative effect, we investigated the Wg expression in *awdi* wing disc cells. Interestingly, the Wg protein is expressed in its correct dorso-ventral domain but shows an altered cellular distribution which impairs its signaling. Further, we show that RNAi-mediated knock down of *awd* in wing discs does not affect Notch signaling. Thus, our analysis of the hypomorphic phenotype arising from *awd* downregulation uncovers a dose-dependent effect of Awd in Notch and Wg signaling.

## 1. Introduction

The *Drosophila abnormal wing disc* (*awd*) gene is the only homolog of *NME1/2* genes identified to date [1,2]. The *NME1* and *NME2* genes belong to a family of ten related genes in humans that are evolutionarily conserved [3]. The *NME1* gene, the first identified metastasis suppressor gene [2], and the *NME2* gene are the ones most often implicated in tumor progression. The NME proteins have several biochemical functions and their tumorigenic role is far to be completely understood. Metastasis suppression was found in a variety of tumor types including melanoma, breast, ovarian, colon and hepatocellular carcinomas [4]. In these malignancies, *NME* expression is inversely correlated with poor survival and tumor grade. On the contrary, in neuroblastoma and hematological malignancies, *NME* expression is indicative of a poor patient prognosis [5,6]. Moreover, extracellular NME proteins have been detected in the medium of cell lines and in human body fluids where their levels vary in different pathological conditions [7]. The physiological function of the secreted NME proteins is still a matter of debate.

Genetic studies on Awd confirmed the multifunctional nature of this protein that fulfils numerous molecular and cellular functions [8]. Awd endocytic function in different tissues is crucial in modulating internalization of signaling receptors and in intracellular trafficking of Notch [9,10,11]. Consistent with the high degree of functional conservation between Awd and its mammalian counterparts, recent studies have shown a role for the NME1/2 proteins in vesicular transport [12]. NME1 is known to inhibit the migratory and invasive potential of cancer cells. It has been shown that the endocytic function of Awd and its human and *C. elegans* homologues is involved in the motility of migrating cells. A dosage-dependent effect of Awd/NDK-1 (Nucleoside Diphosphate Kinase-1) has been reported on the migration of tracheal and border cells in *Drosophila* and of distal tip cells in *C. elegans* [13]. Furthermore, a role in the maintenance of genomic stability has also been assigned to both NME1 and Awd [14,15]. In our previous analyses we found that in larval wing disc mitotic clones lacking the Awd function Notch signaling was halted, and the expression of its target genes, including the signaling molecule Wg, was blocked [10,16]. RNA interference (RNAi)-mediated *awd* (*awdi*) gene silencing in the wing disc posterior compartment allowed us to uncover the *awd* role in maintenance of genomic stability [15]. RNAi-mediated *awd* silencing led to CIN (Chromosomal Instability) and to JNK (Jun amino-terminal kinases)-mediated cell death of larval wing disc cells. Furthermore, block of apoptosis of *awdi* CIN cells resulted in upregulation of MMP1 (Matrix Metalloproteinase 1), cell movement toward the basal side of the epithelium, and aneuploidy. The forced survival of *awdi* CIN cells did not lead to wing disc hyperplasia, differently from the overgrowth arising from forced survival of CIN cells lacking the function of spindle assembly checkpoint (SAC) genes [15,17].

Unrepaired DNA damage is deemed as a contributor to major genome rearrangements including aneuploidy. Double-strand DNA breaks (DSBs) are among the most severe DNA lesions that lead to the cellular DNA damage response (DDR) to repair genomic lesions. The DDR activates the p53 tumor suppressor that in turn promotes the expression of proteins involved in multiple processes including DNA repair and apoptosis [18].

Herein, we further study the cellular effects induced by *awd* silencing. We find that the block of p53 activity in *awdi* wing disc cells does not enhance apoptotic cell death suggesting that *awdi* does not induce the p53-mediated response to repair DNA damage. The results of our analyses also show that, in contrast with *awd* where complete loss of function that halts Notch signaling and blocks Wg expression, *awdi* does not perturb Notch signaling but alters Wg distribution and signaling fully accounting for the lack of wing disc overgrowth. Thus, the present analysis of the hypomorphic phenotype arising from *awd* downregulation uncovers a dose-dependent effect of Awd in Notch and Wg signaling.

## 2. Results

### 2.1. Effects of Awd Silencing in the Larval Wing Disc

**Figure 1:**
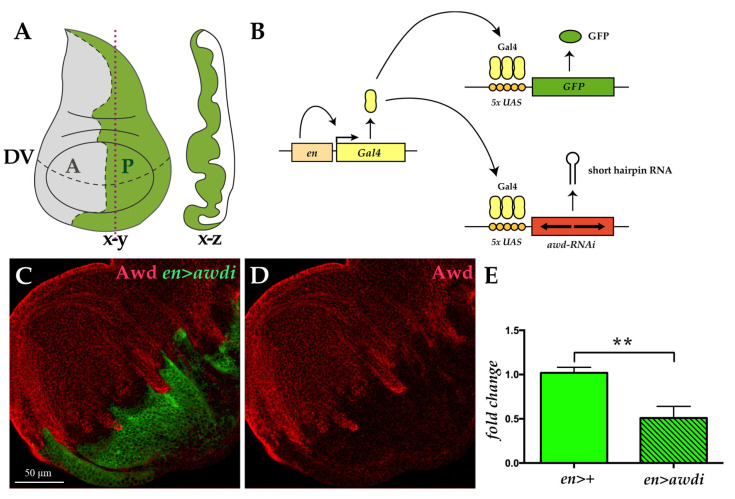
Transgenic RNA interference (RNAi) approach downregulates Awd. (**A**) The wing disc, like the other imaginal discs, is a sac-like structure (x-z cross section along the vertical plane indicated by red dots) composed of a single layered epithelium whose morphology matures during larval development (x-y frontal section). The dashed lines mark the boundary between anterior (A, grey) and posterior (P, green) compartments as well as the dorso-ventral (DV) boundary. (**B**) Illustration of the *UAS/Gal4* expression system applied to drive targeted coexpression of the *UAS-awdi* and *UAS*-*GFP* transgenes. (**C**,**D**) Confocal microscopy image of wing disc dissected from *en>awdi* larvae and immunostained for Awd (red in **C**,**D**). (**E**) qRT-PCR analysis of *awd* transcript level in cephalic complexes and wing discs dissected from larvae of the reported genotypes. Graphs represent mean ± SD; *n* = 3; ** = *p* < 0.01.

The *Drosophila* larval development is characterized by the progression of timed developmental stages. After larval hatching, larvae go through three instar stages separated by moults, followed by pupariation and metamorphosis, giving rise to the adults. We analyzed the effect of *awd* downregulation in the wing disc, the primordium of *Drosophila* adult wing, which is a powerful model for studying tumorigenic growth [19] (Figure 1A). The wing disc is composed of undifferentiated cells that divide throughout larval development before undergoing metamorphosis during pupal stage to form the wing and part of the thoracic wall of the adult fly.

We applied the *UAS/Gal4* system to downregulate in vivo *awd* gene expression by transgene-mediated RNA interference (RNAi) [20] (Figure 1B). To mark cells with downregulated Awd level we coexpressed the *UAS-awd* interfering transgene (*awdi*) and the GFP marker. The *engrailed-Gal4* (*en-Gal4*) enhancer trap line has been used to control expression of both transgenes. This line expresses the Gal4 transcriptional activator within the posterior compartment of developing tissues [21]. Using the *en-Gal4 driver* we downregulated Awd expression throughout development and analyzed the cellular phenotype arising from Awd silencing in the wing disc. Given the posterior compartment specificity of the *en-Gal4* driver, in each wing disc from *en-Gal4*, *UAS-GFP*; *UAS-awdi* (hereafter abbreviated *en>awdi*) larvae, the posterior domain will be composed by cells undergoing Awd downregulation (GFP-positive) while the adjacent anterior domain containing normal cells will serve as control (GFP-negative) (Figure 1A,B). Successful knockdown of Awd was observed as seen by the reduced Awd protein level in the posterior compartment of *en>awdi* wing disc (Figure 1C,D). Consistent with this data, a significant decrease of *awd* transcript (Figure 1E) was detected by quantitative RT-PCR (qRT-PCR) analysis of wing disc and cephalic complexes expressing *awdi* in the posterior compartment under control of *en-Gal4*.

**Figure 2:**
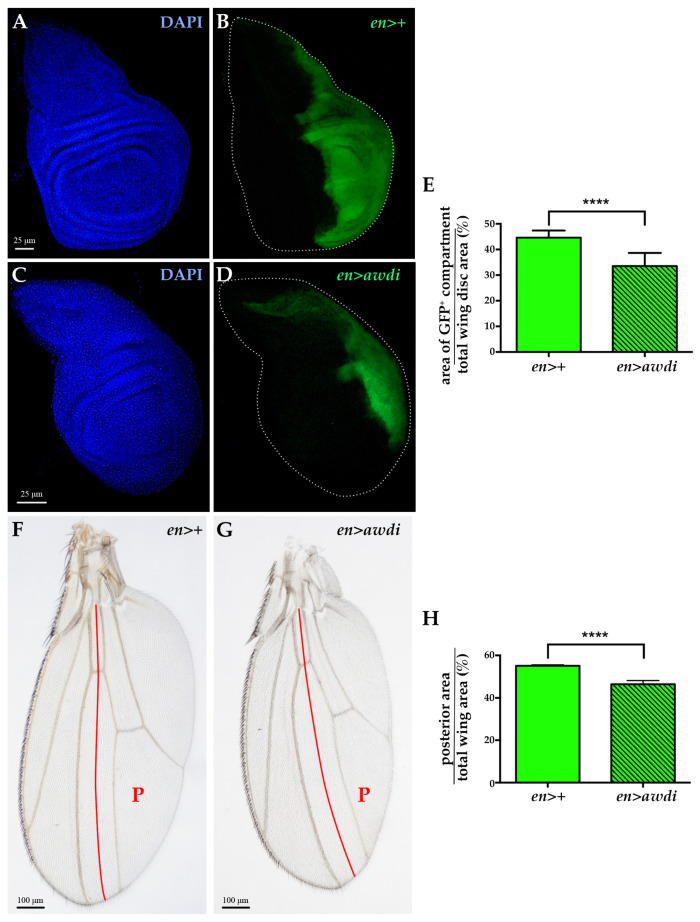
Posterior compartment reduction in *en>awdi* wing disc and adult wing. (**A**–**D**) Fluorescence microscopy image of wing discs dissected from *en>+* (**A**,**B**) and *en>awdi* (**C**,**D**) larvae. Wing discs are labelled by DAPI (**A**,**C**) and GFP (**B**–**D**). Quantification of the average of the posterior area (GFP)/total area ratio in wing discs (**E**). Light micrographs of adult wings dissected from *en>+* (**F**) and *en>awdi* (**G**) males. Quantification of the average of the posterior area (GFP)/total area ratio in adult wings (**H**). The red line marks the boundary between the anterior and posterior (P) compartments. The graphs represent the mean ± SD; **** = *p* < 0.0001.

We have previously reported that *awd* silencing in the posterior domain induces CIN without hyperproliferative effect [15]. In the present study, we quantified the ratio between the posterior domain area and the total area of both *en>awdi* wing discs (*n* = 10) and control *en>+* wing discs (*n* = 10), marked by GFP expression. The results showed a statistically significant reduction (Figure 2E) of the posterior domain of *en>awdi* wing disc (Figure 2C,D) in comparison with the same domain of control wing disc (Figure 2A,B). We extended our analysis to the wing of *en>awdi* adults which shows defects in the posterior region [15]. The quantification of the ratio between the posterior domain area and total wing area showed a statistically significant reduction of the posterior domain (Figure 2F–H).

### 2.2. awdi Induced Cell Death is p53 Independent

**Figure 3:**
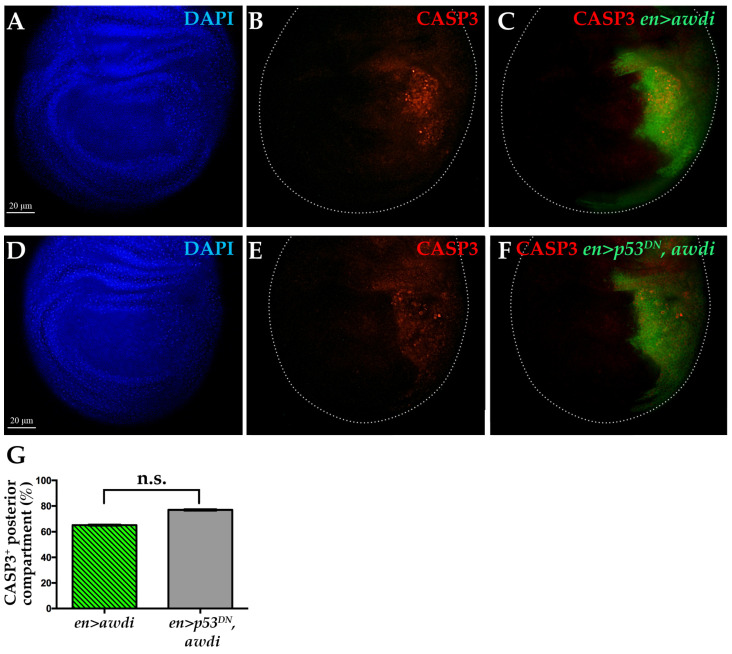
Caspase 3 activation following *awdi* is unaffected by coexpression of p53^DN^. Fluorescence microscopy image of wing disc dissected from *en>awdi* (**A**–**C**) and *en>p53^DN^*, *awdi* (**D**–**F**) larvae. Wing discs are labelled by DAPI (**A**,**D**), anti-activated Casp3 (**B**,**C**,**E**,**F**) and GFP (**C**–**F**). White dots outline wing discs. Quantification of the CASP3-positive wing discs (**G**). The graphs represent the mean ± SD; n.s.: not statistically significant.

p53 activity is necessary to repair IR-induced DSBs in wing disc [22]. Furthermore, IR-induced DSBs lead to JNK-mediated apoptotic cell death [23]. It has also been reported that chromosomal instability leads to p53-independent cell death [17,24]. Therefore, we analyzed apoptosis in *en>awdi* wing disc lacking p53 activity. We coexpressed *awdi* and the transgene coding for the H159N dominant negative form of p53 (*p53^DN^*) [25] in the posterior domain of wing disc. Then, we compared apoptosis in *en>awdi* and *en>p53^DN^*, *awdi* by looking at Casp3 activation. Up to 65% of *en>awdi* wing discs (*n* = 23) showed activated-Caspase 3 signal in the posterior domain, confirming our previous results [15] (Figure 3A–C,G). The coexpression of *awdi* and *p53^DN^* transgenes resulted in 77% wing discs (*n* = 26) with activated-Caspase 3 in the posterior domain (Figure 3D–F,G). Thus, block of the p53 function does not modify the occurrence of apoptosis in *en>awdi* wing discs showing that *awdi* leads to p53-independent cell death. This result further suggests that *awdi* does not trigger the p53 activity of DSB repair.

### 2.3. Awd Gene Function is Required for Wg/Wnt Signaling

**Figure 4:**
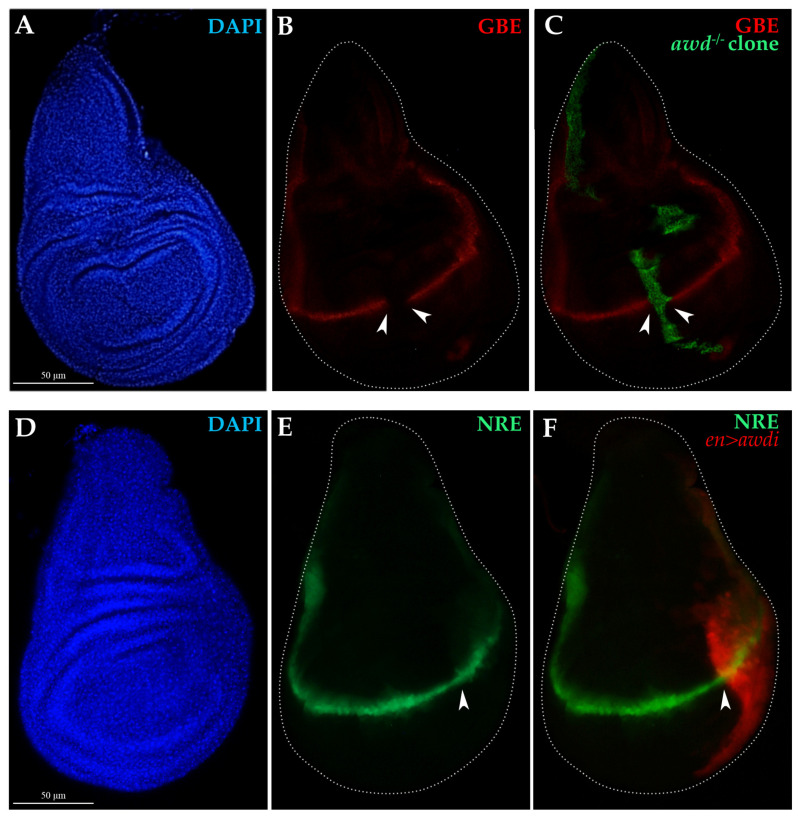
Analysis of Notch signaling in *awd^−/−^* clones and *en>awdi* wing discs. (**A**–**C**) Fluorescence microscopy image of wing disc dissected from larvae carrying *awd^J2A4^* MARCM clones (*awd^−/−^*) marked by GFP expression (**C**) and the *GBE-lacZ* transcriptional reporter for Notch activity. Wing discs are labelled by DAPI (**A**), anti-β-Gal (**B**,**C**) and GFP (**C**). (**D**–**F**) Fluorescence microscopy image of wing disc dissected from *en>awdi* larvae carrying the *NRE-eGFP* (**E**,**F**) transcriptional reporter for Notch activity. Wing discs are labelled by DAPI (**D**) and RFP marking the posterior domain (**F**). White dots outline wing discs, white arrowheads mark respectively the boundary of the MARCM clone (**B**,**C**) or the boundary of the posterior compartment (**E**,**F**).

Overexpression of the Wg mitogen in aneuploid CIN cells induces over proliferation of surrounding cells in the wing disc [17]. The absence of hyperproliferative effect in tissue depleted of Awd function is consistent with our previous findings obtained using the null *awd^J2A4^* allele, in which the Notch signaling is impaired and blocks Wg expression at the wing disc DV boundary [10]. To monitor Notch signaling we used reporter constructs carrying either the *lacZ* or *GFP* reporter genes under control of regulatory elements from Notch-responsive genes (see Section 4.1). The expression of the *GBE-lacZ* (*GBE*) reporter for Notch activity [26] is absent in clones of cells homozygous for the *awd^J2A4^* allele (Figure 4A–C) [10]. Since RNAi often leads to incomplete loss of gene function [27], it seemed interesting to analyze the Notch and Wg signaling pathways in *en>awdi* discs. The expression of the *NRE-eGFP* transcriptional reporter for Notch activity [28] is unaltered at the dorso-ventral boundary of the wing disc showing that the Notch signaling is active in the posterior compartment of all the *en>awdi* wing discs (*n* = 24). (Figure 4D–F).

**Figure 5:**
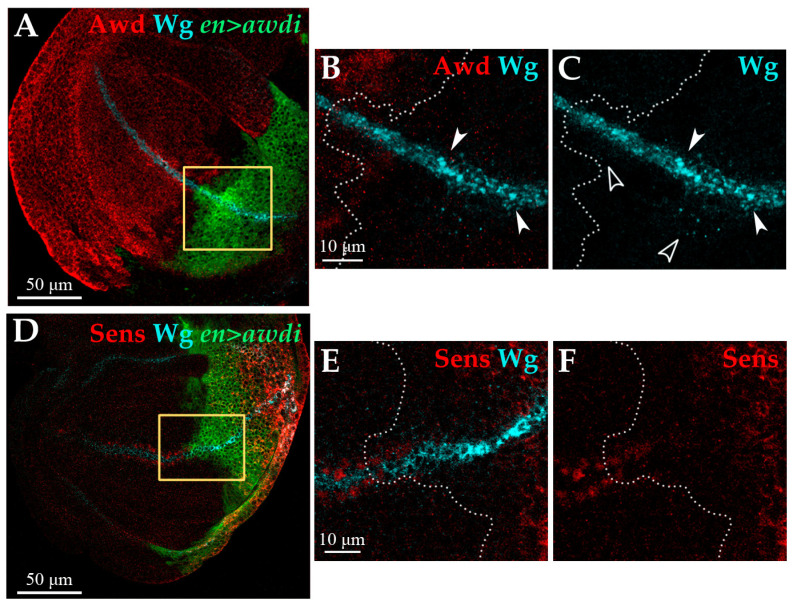
*awdi* impairs Wg signaling. Confocal microscopy images of wing discs dissected from *en>awdi* larvae. Wing discs are labelled by anti-Awd (**A**,**B**), anti-Wg (**A**–**E**), anti-Sens (**D**,**F**). Yellow boxes in (**A**,**D**) outline the enlarged area showed in (**B**,**C**,**E**,**F**) respectively. White dots outline the boundary between anterior and posterior compartments (**B**,**C**,**E**,**F**). White arrowheads mark large aggregates of Wg protein, empty arrowheads mark Wg spots (**B**,**C**).

Consistent with this result, we observed that the Wg protein is expressed in the DV boundary of anterior and posterior compartments of *en>awdi* wing discs but in these cells the Wg protein had an altered distribution (Figure 5A–C). The comparison between anterior and posterior Wg-producing cells clearly showed a marked accumulation in large aggregates of the Wg protein in the latter (Figure 5B,C, arrowheads). Besides Wg-producing cells, the Wg protein is detected also in Wg-receiving cells where it forms spots whose intensity and number decrease with distance from the Wg-producing cells [29]. This punctate staining is well evident in the posterior compartment of *en>awdi* disc showing Wg secretion and gradient formation (Figure 5C, empty arrowheads). In order to assess Wg activity, we analyzed the expression of *senseless* (*sens*), a Wg short-range target gene [30]. In wild type wing discs Sens is expressed in two stripes of signal-receiving cells flanking the Wg-expressing cells at the DV boundary. In the posterior domain of *en>awdi* wing disc, the expression of *sens* in signal-receiving cells abutting Wg-expressing cells is lost (Figure 5D,F) indicating the loss of Wg short-range signaling. Unexpectedly, ectopic Sens expression is detected in the posterior domain far from DV Wg-producing cells.

These results indicate that RNAi-mediated *awd* silencing driven by *en-Gal4* only partially suppresses *awd* gene function leading to a hypomorphic phenotype. Indeed, 23.48% of *en>awdi* survived to adult stage (*n* = 132). To further confirm the hypomorphic effect of *en>awdi* we compared the ubiquitous *awd* silencing phenotype to the one of homozygous null *awd^J2A4^* mutant. The *awd^J2A4^* mutation is lethal recessive [31]. We found that ubiquitous silencing of *awd* driven by the *tub-Gal4* also caused lethality of *tub>awdi* flies. Wild type larval development proceeds through two different stages before reaching the third instar stage at about 120 h after egg laying at 25 °C. During late third instar the larvae start crawling and pupariate. To examine larval viability, we transferred third instar larvae onto fresh food and waited for them to complete development. While all *awd^J2A4^* null larvae failed to pupariate and die (*n* = 120), 64.9% of *tub>awdi* larvae (*n* = 111) reached the pupal stage. Thus, the ubiquitous *awd* silencing allows development to proceed further confirming that RNAi mediated *awd* silencing results in a reduction of *awd* level.

## 3. Discussion

In the present study, we have analyzed the effect of *awd* downregulation driven by RNAi in the wing imaginal disc model. We have already shown that reduction of *awd* function in the posterior compartment of wing disc causes genomic instability and that forced survival of *awdi* CIN cells does not lead to overgrowth of the wing disc [15]. Our present data indicate that reduced *awd* gene expression results in a hypomorphic phenotype that leads to p53-independent cell death. Furthermore, we show that *awdi* halts the Wg signaling pathway in a wing disc fully accounting for the lack of wing disc overgrowth. In contrast with *awd* complete loss of function that affects Notch signaling and blocks Wg expression, the *awdi* hypomorph shows unperturbed Notch signaling and altered distribution of Wg protein at the DV boundary of wing disc. Thus, the study of *awdi* hypomorphic effect advanced our knowledge of Awd function indicating for the first time that Awd is involved in Wg signaling.

Awd has a well-known endocytic function that is required in multiple tissues for many growth-factor-mediated signaling pathways [8]. Wg distribution in *awdi* cells at the DV boundary of the wing disc clearly indicates altered Wg trafficking that blocks expression of the *sens* short-range target gene.

The Wg morphogen acts at short- and long-range distances thanks to different transport mechanisms. The molecular events underlying Wg secretion are partially known but a key role emerged for endocytosis in both short and long range Wg signaling.

**Figure 6:**
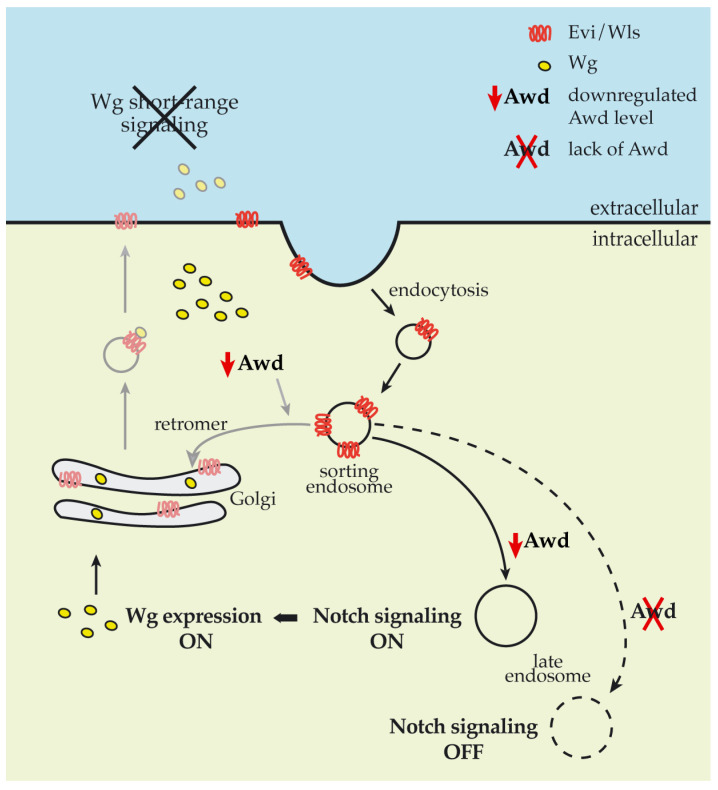
Impact of Awd levels on Notch and Wg signaling pathways. Schematic showing the impact of reduced (solid lines) and null (dashed line) Awd levels in cells at the DV boundary of wing disc. Grey lines show working hypothesis on the Awd involvement in recycling back the Evi chaperonin.

Short-range signaling mainly occurs via apical secretion of Wg from the Wg-producing cell [32]. Evenness interrupted/Wintless (Evi/Wls) and p24/Opium chaperonins are involved in the ER to PM transport of Wg [33,34]. After releasing Wg in the extracellular space, Evi/Wls is endocytosed by the Wg-producing cell and undergoes a retromer-dependent retrograde transport to the trans Golgi network (TGN) [34,35]. Loss of function of the retromer component Vps35 in the wing disc results in reduced Wg secretion and in loss of expression of the short-range target *sens* while it leaves unaltered the expression of the long-range target *distalless* [36]. In summary, endocytosis of Evi/Wls chaperonin, and subsequent retrograde transport to TGN, is a key step in short-range Wg signaling. Therefore, we suggest that the Awd endocytic function could be involved in recycling back the Evi chaperonin to the Wg-producing cell (Figure 6).

Endocytosis in Wg-producing cells also plays an additional role since loss of *shi*/*dyn* function results in accumulation of Wg protein in Wg-producing cells at the DV boundary of wing discs [29]. Further analysis of Wg trafficking showed that Shi/Dyn-mediated endocytosis from the apical surface is a key step in Wg transcytosis [37]. Wg is secreted from the apical surface where it activates short-range signaling. After Shi/Dyn-mediated endocytosis, Wg undergoes the endosomal route and is transported to the basolateral membrane to be secreted and form the extracellular gradient that activates long-range target genes.

Our analysis of Wg protein distribution in *awdi* wing disc cells showed its accumulation in cells at the DV boundary and the lack of its short-range signaling as shown by the loss of *sens* expression in cells flanking the Wg-expressing cells. The presence of spots in neighboring cells indicates that the Wg protein travels at a distance from the DV boundary and forms its gradient. We suggest that this fraction of Wg protein could be responsible for the ectopic expression of Sens detected in the posterior compartment of the *en>awdi* wing disc. This is an interesting finding, which warrants further investigation.

In addition, Wnt signaling is involved in the regulation of microtubule stability and orientation of the mitotic spindle [38]. The Wnt pathway component β-catenin and its interacting proteins localize at the centrosome and their function is required for mitotic progression. Recently, work in *Drosophila* neurons showed that Wnt signaling proteins play a key role in controlling polarity and nucleation of dendritic microtubules [39]. Since these Wnt signaling proteins colocalize with Rab5, it has been suggested that they are located on early endosomes. An interesting possibility could be that impaired Wg signaling contributes to the aneuploidy arising from *awdi*.

Our present data showed that absence of Awd and its reduced levels exert an opposing effect on Notch signaling (Figure 6). In a previous work we have already shown Awd requirement in the early step of Notch intracellular trafficking, after the Shi/Dyn-mediated cleavage of membrane invagination, for Rab5 activity in early endosome maturation [10]. Rab5 regulates early steps of endocytosis including endosomal sorting and fusion of endocytic vesicles. Furthermore, Rab5 plays a key role in the assembly of endosomal machinery since its depletion below a critical level leads to the loss of the endolysosomal pathway [40]. Therefore, we suggest that an absent and reduced Awd level impacts different trafficking pathways dependent on Rab5 activity leading to an opposite effect on Notch signaling.

*awd* is the only *NME1/2* homolog gene identified in *Drosophila*. *NME1/2* are metastasis suppressor genes that exhibit oncogenic function in some tumoral context. Furthermore, *NME1/2* expression levels are inversely correlated with patient’s prognosis in different cancer cohorts [8,41]. Interestingly, our data show that different levels of Awd elicit different effect on key signal transduction pathways such as Notch and Wg. Moreover, Wg signaling requires different transport mechanisms that are context- and tissue-specific [42]. Further studies of the cellular effects arising from *awd* downregulation could help in understanding the complex functions played by NME1/2 proteins.

## 4. Materials and Methods

### 4.1. Drosophila Stocks

*Drosophila* stocks were maintained on standard cornmeal/yeast medium under 12:12 h light/dark cycle at 25 °C and crosses were carried out at the same temperature. The following stocks were obtained from Bloomington Drosophila Stock Center: #33712: *y*; *UAS-awd-RNAi/TM3*, *Sb*; #8420: *y*,*w*; *UAS-p53^H159N^*; #5072: *w*; *UAS-p35*; #30564: *y*,*w*, *en-Gal4^e16E^* (*en-Gal4*). The *w*, *FRT82B*, *awd^J2A4^*/*TM6B* was from T. Hsu, (National Central University, Zhongli, Taiwan) and *en-Gal4*, *UAS-GFPmCD8*/*Gla*, *Bc* was from D. Grifoni (University of Bologna, Bologna, Italy). Notch signaling was monitored using the *Gbe*+*Su(H)_m8_-lacZ* from S. Bray (University of Cambridge, Cambridge, UK) [26] and the *NRE-eGFP* #30727 from BDSC. Activation of the Notch pathway in the developing *Drosophila* wing leads to the *Su(H)* (*Suppressor of Hairless*)-dependent expression of target genes like *E(spl)m8* (*Enhancer of Split m8*). GBE and NRE reporters consist of *Su(H)* binding motifs which control reporter gene expression and represent a readout for Notch pathway activity.

Larvae of the following genotypes (1) *en-Gal4*, *UAS-GFPmCD8/+*; *UAS-awdRNAi/+* (2) *en-Gal4*, *UAS-GFPmCD8*/*UAS-p53^H159N^*; *UAS-awdRNAi*/*+* (3) *en-Gal4*, *UAS-GFPmCD8/+* were obtained by crossing the parental strains. To induce *awd^J2A4^* MARCM clones in wing discs, progeny from appropriate crosses was collected for 24 h. At 48 h after egg laying progeny was subjected to 1-hour heat shock treatment at 37 °C and kept at 25 °C to allow further development. *yw*, *hs-flp*/*Gbe+Su(H)_m8_lacZ*; *act-Gal4*, *UAS-GFP*/*+*; *FRT82B*, *awd^J2A4^*/*FRT82B*, *act-Gal80* third instar stage larvae were then collected and subjected to immunohistochemistry experiments. The genotypes of flies and larvae used for the analyses are the following: Figure 1C,D, Figure 2C,D, Figure 3A–C, Figure 5A–F: *en-Gal4*, *UAS-GFPmCD8*/*+*; *UAS-awdi*/*+*. Figure 2A,B: *en-Gal4*, *UAS-GFPmCD8*/*UAS-nGFP*. Figure 3D–F: *en-Gal4*, *UAS-GFPmCD8*/*p53DN*; *UAS-awdi*/*+*. Figure 4A–C: *hs-flp*/*Gbe+Su(H)_m8_lacZ*; *act-Gal4*, *UAS-GFP*/*+*; *FRT82B*, *awd^J2A4^*/*FRT82B*, *act-Gal80*. Figure 4D–F *en-gal4*/*NRE-eGFP*; *UAS-awdi*/+.

### 4.2. Immunofluorescence Microscopy

Larval tissues were collected and treated at 110–120 h after egg laying. Larvae were dissected in 1xPBS at room temperature and fixed for 20 min in 4% formaldehyde and the immunostaining procedure was performed as previously described [43]. The following primary antibodies were used: rabbit anti-Awd (1:2000; [9]); rabbit anti-cleaved-Caspase3 (1:100; Cell Signaling Technology); mouse monoclonal anti-β-gal (1:25; 40-1A, DSHB); mouse monoclonal anti-Wg antibody (1:50; 4D4, DSHB); guinea pig anti-Sens (1:1000; [30]). The following secondary antibodies from Jackson ImmunoResearch Laboratories were used: Cy3-conjugated goat anti-mouse (1:500); Cy3- (1:1000) or DyLight 649- conjugated anti-rabbit IgG (1:500) and Cy3-conjugated guinea pig (1:800). DNA staining was carried out by incubating wing discs for 10 min with 4′,6-diamidino- 2-phenylindole (DAPI; Sigma-Merck) at 0.5 μg/mL in PBS followed by several washes with PBS. The samples were then mounted in Fluoromount-G (Electron Microscopy Sciences, Hatfield, PA, USA) and were subsequently analyzed with conventional epifluorescence on a Nikon Eclipse 90i microscope (Nikon, Japan) and with a TCS SL Leica confocal system (Leica, Germany). Digital images were processed and assembled using Adobe Photoshop software (Adobe, San Jose, CA, USA). No biased image manipulations were applied.

### 4.3. Total RNA Extraction, cDNA Synthesis and Real Time PCR

Ten cephalic complexes which include the brain hemispheres and ventral nerve cord, and the associated imaginal discs including the wing discs were dissected from *en>awdi* and *en>+* control third instar larvae in PBS. Three independent biological replicates were analyzed. Total RNA was extracted using TRI Reagent (Sigma-Merck, Darmstadt, Germany) RNA extraction protocol as described in [44,45]. RNA concentration and purity were estimated by measuring the absorbance at 260 nm and A260/A280 ratio. 250 ng RNA was retro-transcribed with Omniscript RT Kit (Qiagen, Hilden, Germany) following the manufacturer’s protocol in an MJ Research PTC-100 Programmable Thermal Controller (BioRad, Hercules, CA, USA). Relative abundance of *awd* transcripts was determined by real-time PCR using SsoFast EvaGreen Supermix (BioRad, Hercules, CA, USA) according to the manufacturer’s protocol. Real-time RT–PCR was performed in the CFX Connect Real-Time PCR Detection System (BioRad, Hercules, CA, USA) through the BioRad Manager Software Version 3.1. All transcript expression values were normalized to *Rpl32*. Primers used for *Rpl32* transcript were 5′GACGCTTCAAGGGACAGTATCTG3′ and 5′AAACGCGGTTCTGCATGAG3′; primers used for *awd* transcript were 5′TGGTCGCCCTGAAGTTCAC3′ and 5′TGACCACATTCAGACCCTCC3′. Relative quantification followed the 2^−ΔΔct^ method [46].

### 4.4. Measure of Posterior Compartment

The measures of the posterior compartment of wing discs were performed on *en>+* and *en>awdi* larval wing discs dissected and fixed as previously described. The *en>+* and *en>awdi* wings were dissected from adult males, dehydrated in ethanol 100% and then mounted on microscope slides in lactic acid/ethanol (6:5). Images of wing discs and adult wings were captured using a Nikon Eclipse 90i microscope and acquired with a Nikon Digital Sight camera (Nikon, Japan).

For each wing disc the total area (DAPI) and the area occupied by GFP+ cells (*en-Gal4*) were calculated using ImageJ software (National Institutes of Health, Bethesda, MD, USA). Likewise, the total area and the posterior area of the adult wing were defined following conventional morphology using the same software. The ratio between the area of the posterior domain and the total area was calculated.

### 4.5. Statistical Analyses

GraphPad Prism 6 software was used for statistical analysis. Statistical significance was determined based on the unpaired one-tail *t*-test performed on three independent experiments. *p* < 0.05 was considered statistically significant (* = *p* < 0.05; ** = *p* < 0.01. *** = *p* <0.001 and **** = *p* < 0.001). All results are expressed as the mean ± standard deviation (SD).

## Abbreviations

*awdi*	RNA interference (RNAi)-mediated awd silencing
CIN	Chromosomal Instability
DDR	DNA Damage Response
DSBs	Double-Strand DNA Breaks
DV	Dorso-Ventral
ER	Endoplasmic Reticulum
JNK	Jun amino-terminal kinases
MMP1	Matrix Metalloproteinase 1
NDK-1	Nucleoside Diphosphate Kinase-1
SAC	Spindle Assembly Checkpoint
TGN	Trans Golgi Network
